# The Development and Use of an Innovative Laboratory Method for Measuring Arsenic in Drinking Water from Western Bangladesh

**DOI:** 10.1289/ehp.7974

**Published:** 2005-05-19

**Authors:** Seth H. Frisbie, Erika J. Mitchell, Ahmad Zaki Yusuf, Mohammad Yusuf Siddiq, Raul E. Sanchez, Richard Ortega, Donald M. Maynard, Bibudhendra Sarkar

**Affiliations:** 1 Better Life Laboratories, Inc., East Calais, Vermont, USA; 2 Bangladesh Association for Needy Peoples Improvement, Chorhash, Kushtia, Bangladesh; 3 Green Mountain Laboratories, Inc., Montpelier, Vermont, USA; 4 Laboratoire de Chimie Nucléaire Analytique et Bioenvironnementale, Université de Bordeaux, Gradignan, France; 5 Johnson Company, Inc., Montpelier, Vermont, USA; 6 Department of Structural Biology and Biochemistry, Hospital for Sick Children, and; 7 Department of Biochemistry, University of Toronto, Toronto, Ontario, Canada

**Keywords:** arsenic, arsenomolybdate, Bangladesh, chronic arsenic poisoning, drinking water, graphite furnace atomic absorption spectroscopy, silver diethyldithiocarbamate, spectrophotometry

## Abstract

All of Bangladesh’s approximately 10 million drinking-water tube wells must be periodically tested for arsenic. The magnitude of this task and the limited resources of Bangladesh have led to the use of low-cost, semiquantitative field kits that measure As to a relatively high 50 μg/L national drinking water standard. However, there is an urgent need to supplement and ultimately replace these field kits with an inexpensive laboratory method that can measure As to the more protective 10 μg/L World Health Organization (WHO) health-based drinking water guideline. Unfortunately, Bangladesh has limited access to atomic absorption spectrometers or other expensive instruments that can measure As to the WHO guideline of 10 μg/L. In response to this need, an inexpensive and highly sensitive laboratory method for measuring As has been developed. This new method is the only accurate, precise, and safe way to quantify As < 10 μg/L without expensive or highly specialized laboratory equipment. In this method, As is removed from the sample by reduction to arsine gas, collected in an absorber by oxidation to arsenic acid, colorized by a sequential reaction to arsenomolybdate, and quantified by spectrophotometry. We compared this method with the silver diethyldithiocarbamate [AgSCSN(CH_2_CH_3_)_2_] and graphite furnace atomic absorption spectroscopy (GFAAS) methods for measuring As. Our method is more accurate, precise, and environmentally safe than the AgSCSN(CH_2_CH_3_)_2_ method, and it is more accurate and affordable than GFAAS. Finally, this study suggests that Bangladeshis will readily share drinking water with their neighbors to meet the more protective WHO guideline for As of 10 μg/L.

The life expectancy in Bangladesh during the mid-1960s was only 46 years. Many premature deaths resulted from drinking surface water that was contaminated with bacteria causing diarrhea, cholera, typhoid, and other life-threatening diseases (Nyrop et al. 1975). Aid agencies, the Bangladesh government, and private individuals began installing 8–12 million tube wells to prevent these deaths by providing access to microbially safe groundwater for drinking [World Health Organization (WHO) 2003]. By 1995, Bangladesh had 120 million people (Monan 1995), approximately 97% of whom drank tube-well water (WHO 2000), and for a variety of reasons the life expectancy had increased to 55 years (Monan 1995).

Regrettably, this new source of drinking water was not tested for toxic metals. In 1993, Dhaka Community Hospital first diagnosed chronic arsenic poisoning caused by drinking Bangladesh’s groundwater [British Geological Survey (BGS) 1999a]. In 1997, our team produced the first national-scale map of As concentration in Bangladesh’s ground-water [Frisbie et al. 1999; U.S. Agency for International Development (USAID) 1997]. This map showed that 45% of Bangladesh’s area had groundwater with As concentrations greater than the 50 μg/L national standard (Frisbie et al. 1999; USAID 1997). In 2003, a risk assessment estimated that 28 million Bangladeshis were drinking water with As concentrations greater than this national standard (Yu et al. 2003). As a result of this exposure, skin cancer, melanosis, leukomelanosis, keratosis, hyperkeratosis, and nonpitting edema from chronic As poisoning are common in Bangladesh (BGS 1999b; Frisbie et al. 2002). In addition, the rates of bladder cancer, liver cancer, and lung cancer are expected to increase in Bangladesh based on an analysis of death certificates for As exposures in Taiwan (Morales et al. 2000).

Fortunately, our 1997 study (Frisbie et al. 1999; USAID 1997) also suggested that testing and sharing tube wells could rapidly and inexpensively provide drinking water with As concentrations less than the 50 μg/L national standard to 85% of Bangladesh’s population. That is, 85% of Bangladesh’s neighborhoods have at least one tube well that does not require treatment for As removal before drinking. Therefore, the vast majority of Bangladeshis with unsafe water could potentially get safe drinking water from their neighbors (Frisbie et al. 1999; USAID 1997). As a result of this discovery, groundwater testing has become a major component of an overall strategy for providing safe drinking water to the people of Bangladesh. To date, > 1 million of Bangladesh’s approximately 10 million tube wells have been tested for As with easy-to-use, relatively inexpensive, and semiquantitative field kits [UNICEF (United Nations Children’s Fund) 2004]. Tube wells are considered safe and marked with green paint if the As concentration is ≤ 50 μg/L, the national standard. Conversely, tube wells are considered unsafe and marked with red paint if the As concentration is > 50 μg/L. Those with safe water are asked to share with their less fortunate neighbors. In addition to this initial survey, periodic testing of tube wells has been recommended to ensure that the population has continued access to safe drinking water. This periodic testing is prudent because the As concentration in some of Bangladesh’s tube wells has changed dramatically over time (Frisbie et al. 1999; USAID 1997).

This urgent need to periodically test approximately 10 million tube wells and the limited resources of Bangladesh have led to the use of these semiquantitative field kits for measuring As. However, these field kits are not precise because the user must estimate the concentration of As from a color chart, similar to that used for measuring pH with pH paper. There are two commonly used field kits in Bangladesh. The first kit is not sensitive and must be modified to detect As at the 50 μg/L national standard. The second kit is inaccurate and must be modified to avoid underestimating the true concentration of As. After modification, this second kit overestimates the true concentration of As (Geen et al. 2005). As a result of these deficiencies, there is an urgent need to supplement and ultimately replace these field kits with a quantitative, accurate, precise, sensitive, inexpensive, and environmentally safe laboratory method for measuring As in Bangladesh’s drinking water. The development and evaluation of such a laboratory method are reported here for the first time.

This new method uses relatively inexpensive reagents and equipment that are easily obtained in Bangladesh and can be used to measure a wide variety of analytes. By design, it uses the same equipment as the silver diethyldithiocarbamate [AgSCSN(CH_2_CH_3_)_2_] method for measuring As [American Public Health Association (APHA) et al. 1989], which is commonly used in Bangladesh. Therefore, the new method can be readily implemented in Bangladesh, and it does not require expensive equipment. Such resources are rare in Bangladesh; for example, in 1997 there was only one atomic absorption spectrometer in the entire country that was used for the routine analysis of As (USAID 1997). Also, it does not require highly specialized equipment, such as devoted As analyzers, which have relatively high cost and limited utility (USAID 1997).

It is very important to realize that the WHO drinking water guideline for As of 10 μg/L is based on a 6 × 10^−4^ excess lifetime skin cancer risk for human males, which is 60 times higher than the 1 × 10^−5^ factor that is typically used to protect public health (WHO 1996). The WHO (1996) states that the drinking water guideline for As should be 0.17 μg/L based on the risk of death from skin cancer. However, the detection limit for most laboratories is 10 μg/L, which is why the less protective guideline was adopted: “Guideline values are not set at concentrations lower than the detection limit achievable under routine laboratory operating conditions” (WHO 1993, 1998).

Similarly, Bangladesh has limited access to atomic absorption spectrometers or other sophisticated instruments for measuring As and uses a much higher 50 μg/L drinking water standard, largely because of the poor accuracy of the AgSCSN(CH_2_CH_3_)_2_ method (USAID 1997). However, a 50 μg/L drinking water standard fails to protect against death from not only skin cancer but also bladder, liver, and lung cancers. Drinking water with 50 μg/L As may cause one extra death from skin cancer per 500 people and one extra death from bladder, liver, or lung cancer per 300 people (Morales et al. 2000). Therefore, > 150,000 Bangladeshis are expected to die from skin, bladder, liver, or lung cancer caused by drinking water with > 50 μg/L As. More than 120,000 of these lives could be saved if Bangladesh complied with the more protective WHO drinking water guideline for As of 10 μg/L by sharing safe water within affected neighborhoods. The present study suggests that Bangladesh could adopt this more protective 10 μg/L guideline for As if it used the arsenomolybdate method for As measurement.

## Materials and Methods

### Sample Collection, Preservation, Shipment, and Analyses

**Figure 1:**
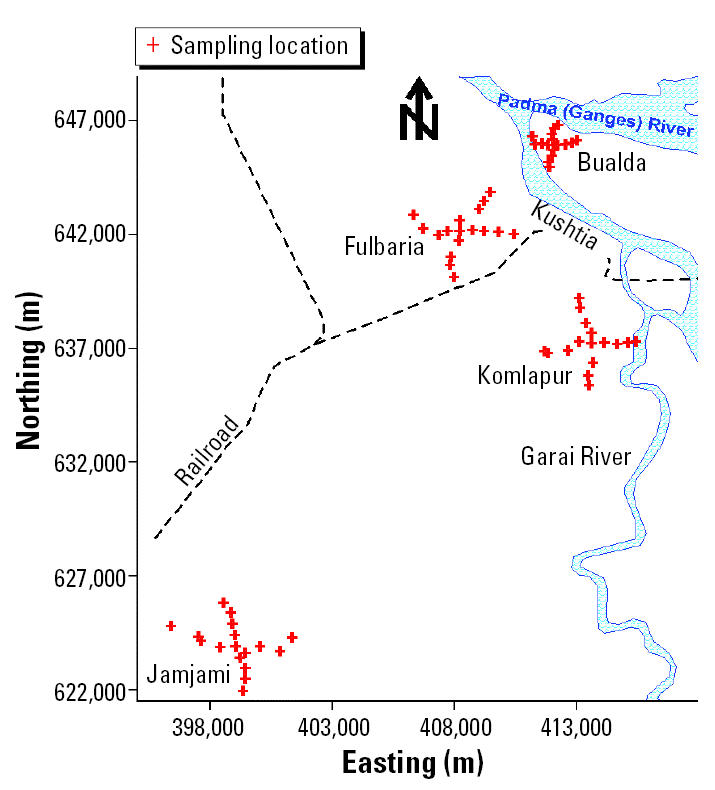
Map of western Bangladesh showing the four neighborhoods where groundwater samples were collected from tube wells. Kushtia is a major city.

Groundwater samples were collected from four neighborhoods in western Bangladesh (Fulbaria, Bualda, Jamjami, and Komlapur) during 18–21 July 2002 (Figure 1). A total of 71 random samples were collected from 67 tube wells in these four neighborhoods. A total of 18 random samples were collected from 17 tube wells in each of three neighborhoods. In the fourth neighborhood, access was denied at 1 sampling location; therefore, a total of 17 random samples were collected from 16 tube wells in that neighborhood.

**Figure 2:**
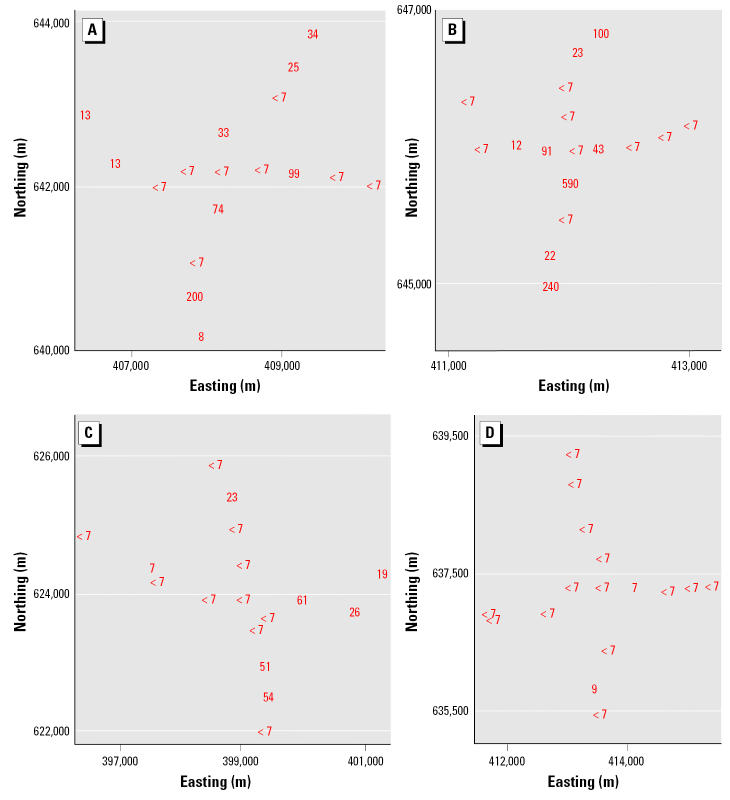
As concentration (μg/L) in tube-well water at each sampling location by the arsenomolybdate method. (*A*) Fulbaria. (*B*) Bualda. (*C*) Jamjami. (*D*) Komlapur.

To the extent possible, the sampled tube wells in each neighborhood were distributed at 500-m intervals along perpendicular axes that radiated in four equal lengths from the center (Figure 2). Two samples were collected from the centermost tube well in each neighborhood. One sample was collected from each of the remaining tube wells. The latitude and longitude of these tube wells were determined using a Garmin Global Positioning System 12 Channel Personal Navigator (Garmin International, Inc., Olathe, KS, USA). The accuracy of this instrument was approximately 15 m.

**Figure 3:**
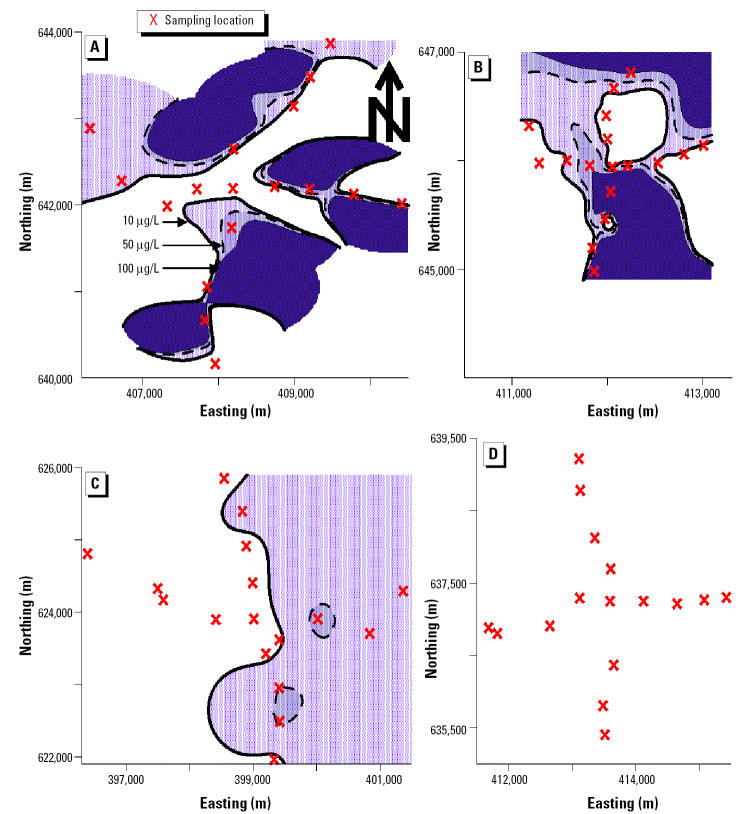
Contour maps of As concentration (μg/L) in tube-well water at each sampling location in the four neighborhoods by the arsenomolybdate method. (*A*) Fulbaria. (*B*) Bualda. (*C*) Jamjami. (*D*) Komlapur. The black contour line represents the 10 μg/L WHO drinking water guideline.

We used established collection, preservation, and storage methodologies to ensure that each sample was representative of groundwater quality (APHA et al. 1995; Stumm and Morgan 1981). Accordingly, all sampled tube wells were purged by pumping vigorously for 10 min immediately before sample collection. All samples were collected directly into polyethylene bottles. These samples were not filtered. Samples were analyzed immediately after collection with pH paper, preserved by acidification to pH < 2 with 5.0 M hydrochloric acid, and stored in ice-packed coolers. The temperature of all stored samples was maintained at 0–4°C until immediately before analysis at laboratories in Dubai and Vermont. In Dubai, all of these samples were analyzed for As by both the arsenomolybdate and AgSCSN(CH_2_CH_3_)_2_ methods. The samples were subsequently shipped to Vermont and analyzed for As by graphite furnace atomic absorption spectroscopy (GFAAS). Contour maps of the As concentration in tube-well water from these four neighborhoods were drawn (Figure 3) as described under “Mapping.”

### Quality Control of Laboratory Analyses

All three methods for determining As were calibrated daily. The calibration for the AgSCSN(CH_2_CH_3_)_2_ method used five different standards, including a blank. The calibrations for the arsenomolybdate and GFAAS methods used six different standards, including blanks. All calibration standards were prepared from the same stock As solution. The concentration of the most dilute nonblank standard was between 1 and 10 times the method detection limit. Calibration check standards were analyzed every 20 samples and as the last analysis of each day to assess data quality. An externally supplied standard was used to assess the accuracy of the calibration standards and calibration check standards (APHA et al. 1995).

We used the recovery of a known addition of standard to eight different samples to assess the matrix effects of all three methods of determining As. In addition, three types of precision were measured for each method from the analysis of seven different standards (precision of standards), the duplicate analyses of eight different samples (precision of samples), and the analysis of a known addition of standard to eight different samples (precision of known additions). The same eight samples were used to assess the matrix effects and precisions of all three methods of determining As (Table 1; APHA et al. 1995).

### Arsenomolybdate Method

#### Apparatus.

**Figure 4:**
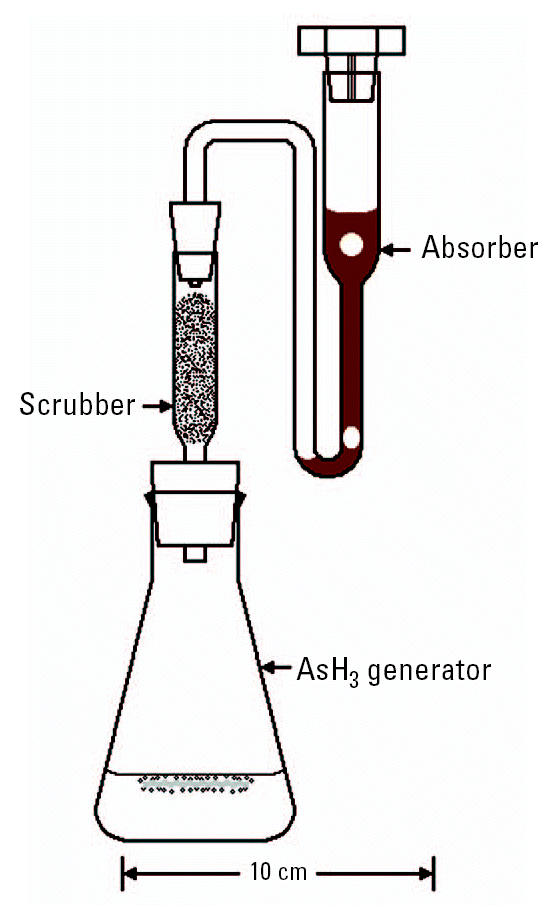
The AsH_3_ generator, scrubber, and absorber used for the two colorimetric determinations of As.

The apparatus, shown in Figure 4, includes an arsine (AsH_3_) generator (a 125 mL Erlenmeyer flask), a scrubber (made from a 10 mL volumetric pipette and a rubber stopper), and an absorber (made from a 20 mL volumetric pipette, a rubber stopper, and a polyethylene cap). The cap of the absorber has four grooves cut along its side to vent H_2_ gas without the loss of liquid during AsH_3_ generation. The spectrophotometer (product no. 6305; Jenway, Essex, UK) was set at 835 nm and used a 1.0 cm glass cell. All glassware was acid washed in 1.00 M HCl.

#### Reagents.

We purchased concentrated sulfuric acid (H_2_SO_4_; product no. 102766H) and concentrated HCl (product no. 101256J) from BDH Laboratory Supplies and zinc (20-mesh granules; product no. 24,346-9) from Aldrich Chemical Company (Milwaukee, WI, USA).

##### As solutions.

We prepared the As solution by dissolving 4.0 g sodium hydroxide (product no. 131687; Panreac Química S.A., Barcelona, Spain) in 10 mL distilled water; arsenic trioxide (1.320 g; product no. 22,762-5; Aldrich) was added, and the solution was diluted to 1,000 mL with distilled water. An intermediate As solution was prepared by diluting 5.00 mL stock As solution to 500 mL with distilled water. This intermediate solution was then used to prepare a standard As solution (10.00 mL intermediate As solution diluted to 100 mL distilled water, which was used in the experiments.

##### Potassium iodide (50.0% wt/vol).

Fifty grams of KI (product no. 102123B; BDH Laboratory Supplies, Poole, UK) was diluted to 100 mL with distilled water and stored in the dark until use.

##### Stannous chloride dihydrate (40.0% wt/vol).

Forty grams of stannous chloride dihydrate (SnCl_2_·2H_2_O; product no. 102704Q; BDH Laboratory Supplies) was dissolved and diluted to 100 mL with concentrated HCl.

##### Lead(II) acetate trihydrate (10.0% wt/vol).

Pb(COOCH_3_)_2_·3H_2_O (10.0 g; product no. 21,590-2; Aldrich) was dissolved and diluted to 100 mL with distilled water.

##### Iodine/KI.

Twenty grams of KI was dissolved in approximately 250 mL distilled water; 12.5 g I_2_ (product no. 20,777-2; Aldrich) was added, and the solution was mixed for several hours using a Teflon-coated magnetic stir bar until all the I_2_ dissolved. The solution was then diluted to 500 mL with distilled water and stored in the dark. The solution was prepared fresh weekly.

##### Sodium bicarbonate (1.00 M).

NaHCO_3_ (product no. 102474V; BDH Laboratory Supplies) was dissolved and diluted to 100 mL with distilled water.

##### Sulfuric acid/ammonium molybdate tetrahydrate.

We prepared 100 mL of 6.50 M H_2_SO_4_ in distilled water. We then diluted 6.9 g (NH_4_)_6_Mo_7_O_24_·4H_2_O (product no. 100282H; BDH Laboratory Supplies) to 100 mL with distilled water. Finally, the 6.50 M H_2_SO_4_ and the 6.9% (wt/vol) (NH_4_)_6_Mo_7_O_24_·4H_2_O were mixed together.

##### Sodium metabisulfite (6.00%wt/vol).

Six grams of Na_2_S_2_O_5_ (product no. 301804E; BDH Laboratory Supplies) were diluted to 100 mL with distilled water; the solution was prepared fresh daily.

##### Stannous chloride dihydrate (0.20% wt/vol).

Fifty milliliters of 40.0% (wt/vol) SnCl_2_·2H_2_O was diluted to 100 mL with distilled water; the solution was prepared fresh daily.

#### Sample treatment.

In order to reduce As(V) to As(III), 35.0 mL of either sample or standard was mixed with 0.35 mL of 50.0% (wt/vol) KI and 0.35 mL of 40.0% (wt/vol) SnCl_2_·2H_2_O in a 125 mL Erlenmeyer flask (to be used as the AsH_3_ generator). The mixture was boiled 1.0 min to reduce As(V) to As(III), and a water bath was used to cool the mixture to room temperature.

#### Scrubber preparation.

The scrubber was prepared by placing 0.17 ± 0.03 g glass wool (product no. 18421; Riedel-de Hafin; Seelze, Germany) onto a piece of Whatman filter paper (Whatman, Kent, UK). Ten drops of 10.0% (wt/vol) Pb(COOCH_3_)_2_·3H_2_O was then added to this piece of glass wool, and the glass wool was squeezed in the filter paper to remove the excess solution. The glass wool was then fluffed and placed in the scrubber (Figure 4).

#### Absorber preparation.

The absorber was prepared by first adding 2.50 mL I_2_/KI solution to a 20 mL test tube; 0.50 mL 1.00 M NaHCO_3_ was then added. This solution was poured into the absorber and the cap was placed on the absorber (Figure 4).

#### Arsine generation, color development, and spectrophotometry.

The amount of time for each step of this procedure, from adding concentrated H_2_SO_4_ to measuring the absorbance, was consistent for all samples and all standards. Two milliliter of concentrated H_2_SO_4_ was mixed with the treated sample or treated standard in the 125-mL Erlenmeyer flask (the AsH_3_ generator); after adding and mixing 10.0 mL concentrated HCl and 1.0 mL 40.0% (wt/vol) SnCl_2_·2H_2_O, 5.0 g Zn was added. The scrubber and absorber were then connected to the AsH_3_ generator (Figure 4). We allowed 30 min for the AsH_3_ to completely evolve from the AsH_3_ generator to the absorber.

The liquid from the absorber was poured into a test tube calibrated to receive 5.00 mL; 0.50 mL of distilled water was then used to rinse the residual liquid from the absorber to the test tube. One milliliter of H_2_SO_4_/(NH_4_)_6_Mo_7_O_24_·4H_2_O solution was added to the test tube and mixed. Then 0.50 mL of 6.00% (wt/vol) Na_2_S_2_O_5_ solution was added to the test tube and mixed. The Na_2_S_2_O_5_ changed the mixture from deep reddish brown to faint yellow (the brown color must be eliminated). When necessary, distilled water was added to until the total volume of liquid was 5.00 mL. Then 0.50 mL 0.20% (wt/vol) SnCl_2_·2H_2_O was added and mixed. After waiting 30 min for the bluish green arsenomolybdate color to develop, we measured the absorbance at 835 nm.

#### Calibration.

For calibration, we added 0, 0.50, 1.00, 2.00, 4.00, and 8.00 mL of standard As solution into six separate 125 mL Erlenmeyer flasks and added distilled water until the total volume of liquid in each flask equaled 35.0 mL (Figure 4). The resulting standards contained 0, 0.50, 1.00, 2.00, 4.00, and 8.00 μg of As or 0, 14, 28.6, 57.1, 114, and 229 μg/L, respectively. We used the sample treatment, scrubber preparation, absorber preparation, AsH_3_ generation, color development, and spectrophotometry procedures described above to analyze these standards. We confirmed that the calibration results follow Beer’s law; that is, we tested for a higher order polynomial relationship to confirm linearity, and we tested the null hypothesis that the y-intercept goes through the origin (Neter et al. 1985).

### Ultraviolet/Visible Spectrum of Arsenomolybdate

In order to determine the absorption maximum of arsenomolybdate we measured the absorption spectra of three arsenomolybdate samples using an Agilent 8453 ultraviolet/visible spectroscopy system (Agilent Technologies, Palo Alto, CA, USA). The wavelengths of these spectra ranged from 190 to 1,100 nm. Each arsenomolybdate sample was prepared from a 229 μg/L As standard solution using the sample treatment, scrubber preparation, absorber preparation, AsH_3_ generation, color development, and spectrophotometry procedures described above under “Arsenomolybdate Method.” The spectrum of each arsenomolybdate sample was measured relative to a blank. Similarly, each blank was prepared from a 0 μg/L As standard solution using these procedures. Each spectrum was measured after 30 min of color development.

### AgSCSN(CH_2_CH_3_)_2_ Method

We analyzed all samples for As following the AgSCSN(CH_2_CH_3_)_2_ method of APHA et al. (1989). In this method, a 125-mL specimen jar was used for the AsH_3_ generator. The scrubber and absorber were identical to those used for the “Arsenomolybdate Method” shown in Figure 4. A 35.0 mL aliquot of sample or standard was placed in the AsH_3_ generator and treated with 5.0 mL concentrated HCl, 2.00 mL 15.0% (wt/vol) KI in distilled H_2_O, and 0.40 mL 40.0% (wt/vol) SnCl_2_·2H_2_O in concentrated HCl to reduce As(V) to As(III). This reduction was allowed 15 min for completion at room temperature. The scrubber was prepared as described above for the “Arsenomolybdate Method.” The absorber received 4.00 mL 0.50% (wt/vol) AgSCSN(CH_2_CH_3_)_2_ in pyridine (C_5_H_5_N). Then, 3.0 g Zn was added to the sample or standard to generate AsH_3_. After 30 min of AsH_3_ generation, the absorbate was measured spectrophotometrically at 535 nm.

### GFAAS Method

All samples were analyzed for As by GFAAS with a Buck Scientific 220AS autosampler, 220GF graphite furnace, and 210VGP atomic absorption spectrometer (Buck Scientific, East Norwalk, CT, USA). A 1.00 mL aliquot of standard As solution, sample, or diluted sample was loaded onto the autosampler. The six standards contained 0, 1.0, 5.0, 15.0, 30.0, and 50.0 μg/L As, respectively. The matrix of each 1.00 mL aliquot was modified with 50.0 μL 10.0% (wt/vol) ammonium nitrate (NH_4_NO_3_; product no. A1216; Spectrum Chemicals and Laboratory Products, New Brunswick, NJ, USA) in deionized water, 50.0 μL 0.2% (wt/vol) palladium nitrate [Pd(NO_3_)_2_] in 2% (wt/vol) HNO_3_ (product no. K, Buck Scientific), and 50.0 μL 1.79% (wt/vol) magnesium nitrate hexahydrate [Mg(NO_3_)_2_·6H_2_O; product no. 5855-1; EM Science, Gibbstown, NJ, USA] in deionized water. The autosampler delivered a 20.0 μL aliquot of this mixture to the graphite furnace. The furnace tube was made from nonpyrolytic graphite (product no. BS300-1253; Buck Scientific). The furnace initialized at 100°C for 10 sec, heated to 250°C for 20 sec, dried the mixture at 250°C for 15 sec, heated to 750°C for 25 sec, ashed the mixture at 750°C for 10 sec, heated to 2,200°C for 1.5 sec, and atomized the mixture at 2,200°C for 3 sec. The sheath and internal flows of argon gas were 1,200 and 200 mL/min, respectively. The absorbance from a hollow-cathode lamp was read at 193.7 nm through a 0.7 nm slit and after deuterium (D_2_) background correction. This absorbance was measured for 2.4 sec during atomization. Finally, this absorbance over time was used to calculate As concentration (Buck Scientific Inc. 2002; Harris 1999).

### Statistics

We measured all 71 samples from this study for As by the arsenomolybdate, AgSCSN (CH_2_CH_3_)_2_, and GFAAS methods. We used a paired *t*-test of the As concentrations from these samples to determine if the arsenomolybdate and AgSCSN(CH_2_CH_3_)_2_ methods gave equivalent or different results (Table 2); a second paired *t*-test to determine if the arsenomolybdate and GFAAS methods gave equivalent or different results (Table 3); and a third paired *t*-test to determine if the AgSCSN(CH_2_CH_3_)_2_ and GFAAS methods gave equivalent or different results (Table 4). Each of these three paired *t*-tests was evaluated at the 95% confidence level (Snedecor and Cochran 1982).

We used an *F*-test to determine if two precision values were equivalent or different. The standard deviation indicates precision (Table 1; APHA et al. 1995), and a variance is indicated by precision squared (Snedecor and Cochran 1982). A ratio of these variances in an *F*-test show the equality of the corresponding precisions (Snedecor and Cochran 1982). Each *F*-test was evaluated at the 95% confidence level.

### Mapping

We used the As concentration by the arsenomolybdate method, the sample location by the Global Positioning System, and the Surfer Surface Mapping System (version 7; Golden Software Inc., Golden, CO, USA) to draw one contour map for each of the four neighborhoods (Figure 3). To map the contours shown in Figure 3, we used a variogram to select the equation that best matched the spatial continuity of the actual As concentrations in each neighborhood (Figure 2). We used inverse-distance weighted least-squares equations (Shepard’s method) for Fulbaria and Bualda and a logarithmic equation for Jamjami. We did not use an equation for Komlapur because all the samples in this neighborhood had As concentrations ≤ 10 μg/L, the WHO drinking water guideline (WHO 1996).

### Societal Evaluation

#### Initial interview during sampling.

One principal user of each tube well was interviewed during groundwater sampling during 18–21 July 2002. Each interview was conducted in Bangla from a list of standard questions. Each interviewee was asked whether alternative drinking water sources were available (rain, ponds, rivers, or canals), and the following information was recorded: *a*) the number of users per tube well; *b*) the depth and age of each tube well; *c*) the results from previous As tests, if any; and *d*) whether any users were known to have melanosis, keratosis, gangrene, skin cancer, conjunctivitis, respiratory distress, or enlarged liver. Finally, the willingness of each interviewee to get safe drinking water from their neighbors or to give safe drinking water to their neighbors was evaluated.

#### Distributing As results.

Our field staff gave a Bangla-language form letter summarizing the neighborhood’s As results to each interviewee 6 months after their groundwater was sampled. In addition, the contents of each letter were explained in Bangla by our field staff. Therefore, each interviewee knew if his tube well had a safe or unsafe concentration of As. Furthermore, each interviewee knew which neighbor’s tube well had a safe or unsafe concentration of As.

Interviewees with As concentrations ≤ 10 μg/L were informed that their tube wells were safe with respect to this element. In addition, they were informed that their tube wells should be tested for As at least once a year because the concentration of As can change over time (Frisbie et al. 1999; USAID 1997). Finally, these interviewees were asked to share their drinking water with those who could not get safe water from their own tube wells.

Interviewees with As concentrations > 10 μg/L (WHO drinking water guideline) were informed that their tube wells were unsafe with respect to this element, and the imminent risk of serious health problems from continuing to drink this water was thoroughly explained. Finally, they were asked to get safe drinking water from their neighbors.

#### Final interview 1 year after sampling.

Each original interviewee, if available, was revisited 1 year after their groundwater was sampled and 6 months after they were given the As results for their neighborhood. If the original interviewee was not available, then a surrogate interviewee was identified. These people were interviewed in Bangla from a list of standard questions. The purposes of this final interview were to determine *a*) if the people with safe tube wells actually gave drinking water to their neighbors who did not have safe drinking water; *b*) whether the people with unsafe tube wells actually got safe drinking water from their neighbors; *c*) whether the people with safe water followed our instructions and retested their tube wells for As; and *d*) the health status of all tube-well users.

## Results and Discussion

### Rationale for sampling drinking water in western Bangladesh.

We chose western Bangladesh for this study because it has some of the widest ranges of groundwater As concentrations in the country, according to our two national-scale surveys (Frisbie et al. 1999, 2002; USAID 1997). This variability is associated with the complex mixture of flood plain deposits that form western Bangladesh (Yu et al. 2003). As a result, the neighborhoods in western Bangladesh usually have at least one tube well that does not require treatment for As removal before drinking (Frisbie et al. 1999; USAID 1997). For example, the As concentrations in our four randomly selected western Bangladeshi neighborhoods ranged from < 0.7 to 590 μg/L, with 30% of samples > 10 μg/L, the WHO drinking water guideline (WHO 1996). This makes western Bangladesh well suited for evaluating our laboratory method of measuring As in drinking water. It is also well suited for determining whether people will use the results from this method to share safe drinking water with their neighbors.

### Independent evaluation of the arsenomolybdate method.

The arsenomolybdate calibration obeys Beer’s law; that is, the plot of absorbance versus As concentration is linear, and the *y*-intercept goes through the origin. A test for a higher order polynomial relationship was done for each daily calibration and routinely confirmed this linearity at the 95% confidence level (Neter et al. 1985). Similarly, a test of the null hypothesis that the *y*-intercept goes through the origin was performed for each daily calibration and routinely confirmed that the *y*-intercept was equivalent to 0, 0 at the 95% confidence level (Neter et al. 1985). The calibration equation from the arsenomolybdate method detection limit study is absorbance = 0.00169 × (micrograms As per liter), and the correlation coefficient (*r*) = 1.00. The slope of the equation and the 7 μg/L method detection limit shown in Table 1 suggest that the colorimetric measurement of As by arsenomolybdate is extremely sensitive. The 101.5 ± 3.6% recovery of known additions shown in Table 1 suggests that the results from the arsenomolybdate method are accurate. The respective 2.1, 4.7, and 7.1 μg/L precisions of standards, samples, and known additions shown in Table 1 suggest that the results from the arsenomolybdate method are reproducible.

In this method, As is removed from the sample by reduction to AsH_3_ gas. This gas is collected in an absorber by oxidation to arsenic acid (H_3_AsO_4_). All previous attempts at this separation have suffered from poor reproducibility because of the incomplete recovery of AsH_3_ (Sandell 1942, 1959). The sources of this incomplete recovery were insufficient concentrations of oxidant in the absorbate, the deterioration of absorbate with time, and improper absorber designs. This problem of incomplete recovery has been solved.

We discovered and corrected an error that has not been reported by previous researchers. This error was from drift caused by the arsenomolybdate absorption spectrum changing with time. Our method requires that the arsenomolybdate color develop for 30 min before its absorbance is measured. This color is relatively stable after 30 min. In addition, a loss of sensitivity from using polychromatic light to measure absorbance has been corrected. Our method requires that the absorbance be measured at 835 nm, the absorption maximum of arsenomolybdate. In addition, previous arsenomolybdate methods were not compared with established methods for measuring As (Milton and Duffield 1942; Sandell 1959). Comparisons of our method with the AgSCSN(CH_2_CH_3_)_2_ and GFAAS methods for measuring As are shown in Tables 1–4.

Finally, all routine analytical methods must use a rigorous quality control plan to identify and correct systematic errors. The quality control plan for the arsenomolybdate method should include daily calibrations and the frequent analysis of blanks, externally supplied standards, calibration check standards, duplicate samples, and known additions of standard to samples (APHA et al. 1995; Frisbie et al. 1999; USAID 1997).

### Arsenomolybdate versus AgSCSN (CH_2_CH_3_)_2_.

We found no difference between the arsenomolybdate and AgSCSN(CH_2_CH_3_)_2_ methods for measuring As at the 95% confidence level, according to a paired *t*-test of all 71 samples from this study (*p*-value = 0.06; Table 2). As a result, these two methods are highly correlated (*r* = 0.993; Table 2). These two methods can quantify As to less than the WHO drinking water guideline of 10 μg/L without expensive or highly specialized laboratory equipment; the cost and required skill to implement these methods are comparable; the precisions of standards for the methods are equivalent at the 95% confidence level; and the precisions of samples for the two methods are equivalent at the 95% confidence level (Table 1).

However, the precision values of known additions for the arsenomolybdate and AgSCSN(CH_2_CH_3_)_2_ methods are different at the 95% confidence level (Table 1). These two precision values were measured using the same eight samples. This suggests that the AgSCSN(CH_2_CH_3_)_2_ method is imprecise at relatively high As concentrations because of the sample matrix. Additional evidence of this imprecision is the 16.6% relative percent difference of the mean As concentrations from all 71 samples measured by the arsenomolybdate and AgSCSN(CH_2_CH_3_)_2_ methods (Table 2). One source of this imprecision may be the incomplete reduction of As(V) to As(III) in Bangladesh’s tube-well water by the relatively mild sample treatment procedure of the AgSCSN(CH_2_CH_3_)_2_ method. More specifically, the arsenomolybdate method uses KI and SnCl_2_·2H_2_O at 100°C for this reduction, whereas the AgSCSN(CH_2_CH_3_)_2_ method uses KI and SnCl_2_·2H_2_O at room temperature. Another source of this imprecision may be the incomplete generation of AsH_3_ in Bangladesh’s tube-well water by the relatively mild AsH_3_ generation procedure of the AgSCSN(CH_2_CH_3_)_2_ method. More specifically, the arsenomolybdate method uses 0.18 g KI, 0.540 g SnCl_2_·2H_2_O, 2.0 mL concentrated H_2_SO_4_, 10.0 mL concentrated HCl, and 5.0 g Zn per 35.0 mL of sample for AsH_3_ generation. In contrast, the AgSCSN(CH_2_CH_3_)_2_ method uses 0.300 g KI, 0.16 g SnCl_2_·2H_2_O, 5.0 mL concentrated HCl, and 3.0 g Zn per 35.0 mL of sample for AsH_3_ generation.

Another important difference between the arsenomolybdate and AgSCSN(CH_2_CH_3_)_2_ methods is related to worker health and environmental protection. More specifically, the arsenomolybdate method uses H_2_O as a solvent, which is nontoxic, nonflammable, and easy to dispose of. In contrast, the AgSCSN(CH_2_CH_3_)_2_ method uses either chloroform (CHCl_3_) or C_5_H_5_N as a solvent (Table 1). All routes of exposure to CHCl_3_ are likely to cause cancer in humans [U.S. Environmental Protection Agency (EPA) 2004]. Moreover, with improper disposal, CHCl_3_ can persist in an aquifer for centuries (Pankow and Cherry 1996). Exposure to C_5_H_5_N may cause increased liver weight and hepatic lesions (U.S. EPA 2004). In addition, C_5_H_5_N is highly flammable and, as a result, presents an acute risk to laboratory workers (Sittig 1985).

In summary, the arsenomolybdate method is more accurate and precise than the AgSCSN(CH_2_CH_3_)_2_ method based on the recoveries of known additions (Tables 1 and 2). The arsenomolybdate method is safer than the AgSCSN(CH_2_CH_3_)_2_ method because it does not use toxic solvents (Table 1).

### Arsenomolybdate versus GFAAS.

We found no difference between the arsenomolybdate and GFAAS methods for measuring As at the 95% confidence level, according to a paired *t*-test of all 71 samples (*p*-value = 0.24; Table 3). As a result, these two methods are highly correlated (*r* = 0.996; Table 3). Both methods can quantify As to less than the 10 μg/L WHO drinking water guideline (WHO 1996; Table 1). However, the equipment cost for the GFAAS method is much greater than that for the arsenomolybdate method (Table 1).

Despite its far lower cost, the arsenomolybdate method is more accurate than the GFAAS method. The recovery of known additions by the arsenomolybdate method is equivalent to 100% (101.5 ± 3.6%; Table 1). In contrast, the recovery of known additions by the GFAAS method is > 100% (103.3 ± 3.1%; Table 1). This suggests that the GFAAS method overestimated the true concentration of As in this matrix by approximately 3.3% (Table 1). This estimate of 3.3% bias by the GFAAS method is based on the recovery of known additions from eight samples. Additional evidence of this bias is the 3.6% relative percent difference of the mean As concentrations from all 71 samples measured by the arsenomolybdate and GFAAS methods (Table 3). This overestimation by the GFAAS method was likely caused by scattered light from sodium chloride or similar matrix salts that remained in the furnace during atomization (Frisbie et al. 1999, 2002; Harris 1999; USAID 1997). If so, the NH_4_NO_3_, Pd(NO_3_)_2_, and Mg(NO_3_)_2_·6H_2_O matrix modifiers and D_2_ background correction did not fully resolve this interference.

In contrast, the GFAAS method is more precise than the arsenomolybdate method. The standards, samples, and known additions are measured with greater precision by the GFAAS method than by the arsenomolybdate method (Table 1). Each of these three *F*-tests was evaluated at the 95% confidence level.

In summary, the arsenomolybdate method is more accurate and affordable than the GFAAS method (Tables 1 and 3).

### AgSCSN(CH_2_CH_3_)_2_ versus GFAAS.

The AgSCSN(CH_2_CH_3_)_2_ and GFAAS methods for measuring As are different at the 95% confidence level, according to a paired *t*-test of all 71 samples (*p*-value = 0.01; Table 4). Furthermore, the As concentrations measured by the GFAAS method were approximately 20.1% greater than those measured by the AgSCSN(CH_2_CH_3_)_2_ method (Table 4). The difference between these methods was likely caused by a combination of two deficiencies: the observed imprecision of the AgSCSN(CH_2_CH_3_)_2_ method at relatively high As concentrations, and the observed bias of the GFAAS method at all As concentrations.

### Societal evaluation.

Any society may accept or reject a given solution to a problem for a variety of reasons. Therefore, it is essential to evaluate the willingness of Bangladeshis to use the results from our method to obtain safe drinking water with As concentrations 10 μg/L from their own wells or from their neighbors, and to give this “safe” drinking water to their neighbors.

Water testing and sharing can provide access to safe drinking water for the millions of Bangladeshis who live in communities where some tube wells are safe and others are not (Frisbie et al. 1999, 2002; USAID 1997). Whether or not this testing and sharing will actually provide safe drinking water in these communities will depend on neighbors correctly understanding the As results for their tube wells and consistently sharing safe water with each other. However, water gathering and use are subject to cultural habits that are difficult to change. The nearly universal switch (97%) in Bangladesh since 1971 from using surface water for drinking to tube-well water has shown that water use traditions can be changed, provided there are extensive community education programs and support from both the government and nongovernmental organizations (WHO 2000).

In Bangladesh, as in most other areas where water must be gathered from a communal source, the chore of water gathering is generally considered to be women’s work. However, Bangladeshi women are constrained by society and their families to stay close to home, whereas unrelated men are generally not welcome inside family compounds. In order for water testing and sharing to be successful as a strategy for providing safe drinking water in Bangladesh, the neighbors as a community must be educated about the meaning of their As results. They must also be willing to use tube wells that may not be close to home or to share their own safe tube wells with unrelated neighbors or strangers.

In our initial survey, which was completed before the owner or regular user of each tube well knew the results from our testing, we asked respondents if they would be willing to use other water sources if their own tube well had unsafe levels of As. We also asked them if they would be willing to share water with their neighbors if their own tube well turned out to be safe. The results were very encouraging; 86% of respondents claimed they would permit family members to gather water from other tube wells if their own tube well had unsafe levels of As, and 94% said they would share water with neighbors if they had safe water and their neighbors did not have safe water.

However, what people say they will do is not always what they actually do in practice. For this reason, we conducted a follow-up survey 6 months after our testing results were distributed to the original respondents or their family members. In this second survey, we learned that almost all tube-well owners (91%) shared water with others. Many respondents noted that sharing water with strangers is customary in village communities and required as a matter of courtesy or charity, which seems to override issues of water safety. Generally, however, after As testing, the status of a tube well becomes known in the community. Once As test results become known in the community, only strangers continue to ask for water from contaminated tube wells. Owners of safe tube wells report that the largest group of non-family members gathering water at their tube wells are neighbors, and 26% of these owners claimed that one reason why they shared their water was because other tube wells had unsafe levels of As and theirs did not.

Instead of sending women out to get water from a stranger’s tube well, men in some families took over this chore. Some men go to public tube wells at mosques to gather water for their families. Others seek water from neighbors’ or relatives’ tube wells. However, this was cited as a source of conflict by 11% of tube-well owners, who did not like having unrelated men enter their family compounds. The location of a tube well is crucial. If the tube well is located on the street, then all may use it freely. In contrast, if the tube well is located inside a family compound, only family members and certain relatives have free access to the compound and the tube well, regardless of the stated willingness of the owner to share the tube well. Distance is also a factor for willingness to gather water from an outside tube well, with one family reporting that it continued to use As-contaminated water because other tube wells were too far away. The maximum distance required to get safe drinking water in each of these four neighborhoods was approximately 0 m in Komlapur, 490 m in Bualda, 1,400 m in Fulbaria, and 2,100 m in Jamjami (Figure 3).

Education programs concerning the dangers of As-contaminated water have achieved some measure of success, because only one owner of an unsafe tube well in our study appeared not to have understood the implications of his tube well’s As results. This owner, a 70-year-old male, professed during the follow-up interview that his water was safe, and he told us that he had continued to use it and share it freely with others.

Many of the tube-well owners (85%) followed our recommendation and had their tube wells retested. This retesting was not performed by our research team. Only one respondent told us that he thought retesting was not necessary (the one who believed his water was safe when it was not); the others who did not retest their water gave cost as the reason for not retesting. People were especially willing to retest their water if this could be done at no cost to them. Of the respondents who had their water retested, 96% reported that the retesting was done with no charge to them; however, two respondents paid to have their water retested.

**Figure 5:**
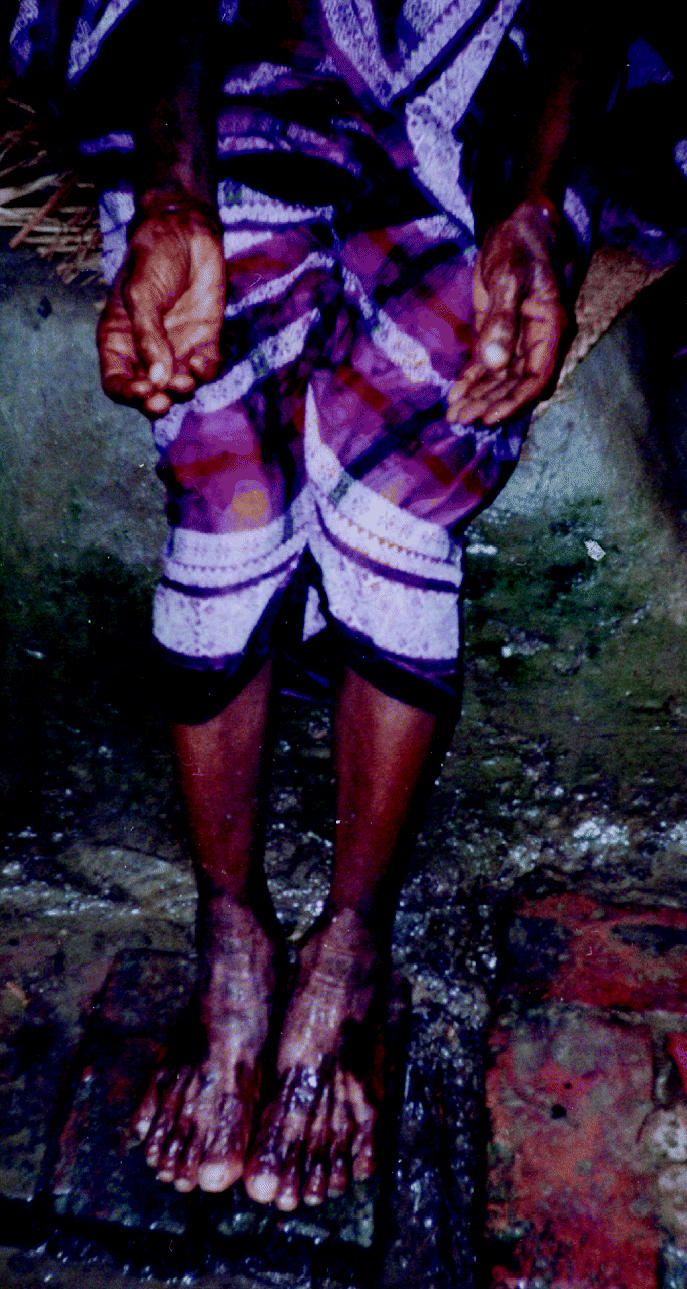
Photograph of a Bangladeshi female with keratosis of the palms and blackfoot disease. Her well contained 1.4 μg/L As on 21 July 2002; she had been drinking from this well for 34 years.

Our survey suggests that the number of tube wells, especially private tube wells, is rapidly increasing in Bangladesh as more families acquire the economic resources to build them. Only 14% of tube wells were reported to have been constructed before 1983 (56% private); 27% were reported to have been constructed from 1983 to 1992 (83% private), and 59% from 1993 to 2002 (92% private), making the tube wells in our random sample from western Bangladesh only 9 years old on average. In addition, we asked whether respondents were aware of any As patients in the area. The number of nearby As patients was not statistically related to the concentration of As during our sampling event (*p* = 0.44). Similarly, the concentration of As during our sampling event was not statistically related to the age of the tube well (*p* = 0.51). In contrast, the number of nearby As patients was statistically related to the age of the tube well and hence the duration of exposure to tube-well water that was potentially contaminated with As. That is, the oldest tube wells were associated with the highest number of nearby As patients (*p* = 0.03). This stresses the fact that duration of exposure to As must be considered in addition to the concentration of As at any given time, because this can explain why the tube wells of some As patients are found to contain very low levels of As but have been used over many years (WHO 2001). For example, Figure 5 shows a female As patient with keratosis of the palms and blackfoot disease. When tested in 2002, the tube well used by this patient had an As concentration of 1.4 μg/L, but she had been drinking from this tube well for 34 years. The As concentration in some of Bangladesh’s tube wells has changed dramatically over time (Frisbie et al. 1999; USAID 1997). These results demonstrate the need to periodically test all drinking water tube wells so that the total lifetime exposure to As can be reduced. Because most of the tube wells in the country have been installed quite recently, the numbers of As patients may begin to increase dramatically as more people develop a history of using tube wells for longer than the 5–10 years it may take to develop symptoms of chronic As poisoning. Finally, as the ages of the tube wells and the length of exposure to As increase, it may become even more vital to adopt a drinking-water standard for As lower than the current Bangladesh standard of 50 μg/L, such as the 10 μg/L WHO guideline (WHO 1996).

## Conclusions

A valid method for the determination of As by arsenomolybdate is now available. This method is more accurate, precise, and environmentally safe than the AgSCSN(CH_2_CH_3_)_2_ method (Tables 1 and 2), and it is more accurate and affordable than the GFAAS method (Tables 1 and 3).

Most important, the arsenomolybdate method is the only accurate, precise, and safe way to quantify As to less than the 10 μg/L WHO drinking water guideline (WHO 1996) without expensive or highly specialized laboratory equipment (Table 1). This suggests that developing countries such as Argentina, Bangladesh, Bolivia, Chile, China, India, Mexico, Nepal, Pakistan, Peru, and Thailand, with limited access per capita to atomic absorption spectrometers or other sophisticated instruments for measuring As, could lower their 50 μg/L drinking water standards to the more protective 10 μg/L guideline if they use the arsenomolybdate method (Bhattacharya et al. 2002; Ng et al. 2003; United Nations 2004). More than 32 million people from these countries drink water with As concentrations greater than their national standards of 50 μg/L (Bhattacharya et al. 2002; Ng et al. 2003; United Nations 2004; Yu et al. 2003). This suggests that > 170,000 people from these countries will die from skin, bladder, liver, or lung cancer caused by drinking water with > 50 μg/L As. If these countries complied with a 10 μg/L drinking water standard for As, perhaps by sharing safe water as was done in the four neighborhoods from this study, > 140,000 of these 170,000 lives could potentially be saved.

In particular, it is very important that the Government of Bangladesh lower its 50 μg/L drinking water standard for As. The rapidly increasing number of tube wells in Bangladesh suggests that the mortality rate from chronic As poisoning is also rapidly increasing. Lowering this standard will reduce exposure and save lives by encouraging the use of safer drinking water.

Finally, our surveys suggest that Bangladeshis will readily test and share their drinking water to meet the more protective WHO guideline for As of 10 μg/L. More specifically, 85% of tube-well owners were concerned enough to retest their water for As within 1 year, and 90% of the tube-well owners that had tube-well As concentrations > 10 μg/L actually gathered water from their neighbors.

## Figures and Tables

**Table 1:** Quality control results, equipment costs, and solvents for the determination of As by arsenomolybdate, AgSCSN(CH_2_CH_3_)_2_, and GFAAS methods.

	Arsenomolybdate	AgSCSN(CH_2_CH_3_)_2_	GFAAS
Method detection limit	7 μg/L	9 μg/L	0.7 μg/L
Equipment cost	$6,700^a^	$6,700^a^	$37,000^b^
Recovery of known additions^c^	101.5 ± 3.6%	103 ± 18%	103.3 ± 3.1%
Precision of standards	2.1 μg/L	2.6 μg/L	0.22 μg/L
Precision of samples	4.7 μg/L	4.5 μg/L	1.7 μg/L
Precision of known additions	7.1 μg/L	24 μg/L	2.0 μg/L
Solvent	H_2_O	CHCl_3_ or C_5_H_5_N	H_2_O

a Includes a spectrophotometer, distillation unit for purifying laboratory water, analytical balance, top-loading balance, hot plate with stirrer, and glassware.

b Includes an atomic absorption spectrometer, distillation unit for purifying laboratory water, analytical balance, top-loading balance, and glassware.

c 95% confidence interval.

**Table 2:** Comparison of As concentrations determined by the arsenomolybdate and AgSCSN(CH_2_CH_3_)_2_ methods.

Measure	Result
Calculated *t*	1.88
Critical two-tailed *t* at α = 0.05 (70 df)	1.99
*p*-Value of paired *t*-test	0.06
Correlation coefficient, *r*	0.993
Mean As concentration by arsenomolybdate	27.6 μg/L
Mean As concentration by AgSCSN(CH_2_CH_3_)_2_	23.4 μg/L
Relative percent difference of these means	16.6%

**Table 3:** Comparison of As concentrations determined by the arsenomolybdate and GFAAS methods.

Measure	Result
Calculated *t*	1.19
Critical two-tailed *t* at α = 0.05 (70 df)	1.99
*p*-Value of paired *t*-test	0.24
Correlation coefficient, *r*	0.996
Mean As concentration by arsenomolybdate	27.6 μg/L
Mean As concentration by GFAAS	28.6 μg/L
Relative percent difference of these means	3.6%

**Table 4:** Comparison of As concentrations determined by the AgSCSN(CH_2_CH_3_)_2_ and GFAAS methods.

Measure	Result
Calculated *t*	2.63
Critical two-tailed *t* at α = 0.05 (70 df)	1.99
*p*-Value of paired *t*-test	0.01
Correlation coefficient, *r*	0.995
Mean As concentration by AgSCSN(CH_2_CH_3_)_2_	23.4 μg/L
Mean As concentration by GFAAS	28.6 μg/L
Relative percent difference of these means	20.1%

## References

[b1-ehp0113-001196] APHA, American Water Works Association, Water Pollution Control Federation 1989. Standard Methods for the Examination of Water and Wastewater. 17th ed. Washington, DC:American Public Health Association.

[b2-ehp0113-001196] APHA, American Water Works Association, Water Environment Federation 1995. Standard Methods for the Examination of Water and Wastewater. 19th ed. Washington, DC:American Public Health Association.

[b3-ehp0113-001196] BGS 1999a. Groundwater Studies for Arsenic Contamination in Bangladesh, Main Report. Nottingham, UK:British Geological Survey.

[b4-ehp0113-001196] BGS 1999b. Groundwater Studies for Arsenic Contamination in Bangladesh, Vol S5. Nottingham, UK:British Geological Survey.

[b5-ehp0113-001196] Bhattacharya P Jacks G Frisbie SH Smith E Naidu R Sarkar B 2002. Arsenic in the environment: a global perspective. In: Heavy Metals in the Environment (Sarkar B, ed). New York:Marcel Dekker Inc., 147–215.

[b6-ehp0113-001196] Buck Scientific, Inc 2002. Buck Scientific 210VGP Atomic Absorption Spectrophotometer Operator’s Manual. East Norwalk, CT:Buck Scientific, Inc.

[b7-ehp0113-001196] Frisbie SH Maynard DM Hoque BA 1999. The nature and extent of arsenic-affected drinking water in Bangladesh. In: Metals and Genetics (Sarkar B, ed). New York:Plenum, 67–85.

[b8-ehp0113-001196] Frisbie SH, Ortega R, Maynard DM, Sarkar B (2002). The concentrations of arsenic and other toxic elements in Bangladesh’s drinking water. Environ Health Perspect.

[b9-ehp0113-001196] Geen AV, Cheng Z, Seddique AA, Hoque MA, Gelman A, Graziano JH (2005). Reliability of a commercial kit to test groundwater for arsenic in Bangladesh. Environ Sci Technol.

[b10-ehp0113-001196] Harris DC 1999. Quantitative Chemical Analysis. 5th ed. New York:W.H. Freeman.

[b11-ehp0113-001196] Milton R, Duffield WD (1942). Determination of arsenic in soils, foods, organic compounds, etc. Analyst.

[b12-ehp0113-001196] Monan J 1995. Bangladesh: the Strength to Succeed. Oxford, UK:Oxfam.

[b13-ehp0113-001196] Morales KH, Ryan L, Kuo TL, Wu MM, Chen CJ (2000). Risk of internal cancers from arsenic in drinking water. Environ Health Perspect.

[b14-ehp0113-001196] Neter J Wasserman W Kutner MH 1985. Applied Linear Statistical Models. 2nd ed. Homewood, IL:Irwin.

[b15-ehp0113-001196] Ng JC, Wang J, Shraim A (2003). A global health problem caused by arsenic from natural sources. Chemosphere.

[b16-ehp0113-001196] Nyrop RF Benderly BL Conn CC Cover WW Eglin DR 1975. Area Handbook of Bangladesh. Washington, DC:American University.

[b17-ehp0113-001196] Pankow JF Cherry JA 1996. Dense Chlorinated Solvents and Other DNAPLs in Groundwater. Portland, OR:Waterloo Press.

[b18-ehp0113-001196] Sandell EB (1942). Colorimetric microdetermination of arsenic after evolution as arsine. Ind Eng Chem.

[b19-ehp0113-001196] Sandell EB 1959. Colorimetric Determination of Traces of Metals. 3rd ed. New York:Interscience Publishers, Inc.

[b20-ehp0113-001196] Sittig M 1985. Handbook of Toxic and Hazardous Chemicals and Carcinogens. 2nd ed. Park Ridge, NJ:Noyes Publications.

[b21-ehp0113-001196] Snedecor GW Cochran WG 1982. Statistical Methods. 7th ed. Ames, IA:Iowa University Press.

[b22-ehp0113-001196] Stumm W Morgan JJ 1981. Aquatic Chemistry. New York:John Wiley & Sons.

[b23-ehp0113-001196] United Nations 2004. United Nations Children’s Fund and Pakistan Council of Research in Water Resources Collaborating to Counter Arsenic Contamination. Available: http://www.un.org.pk/unic/newsletters/NEWSLETTER040309.htm [accessed 24 March 2004].

[b24-ehp0113-001196] UNICEF 2004. Summary of Midterm Reviews and Major Evaluations of Country Programmes. Available: http://www.unicef.org/about/exeboard/files/04-PL31.pdf [accessed 19 November 2004].

[b25-ehp0113-001196] USAID 1997. Report of the Impact of the Bangladesh Rural Electrification Program on Groundwater Quality. Dhaka, Bangladesh:U.S. Agency for International Development.

[b26-ehp0113-001196] U.S. EPA (United States Environmental Protection Agency) 2004. Integrated Risk Information System. Available: http://www.epa.gov/iris/ [accessed 1 April 2004].

[b27-ehp0113-001196] WHO 1993. Guidelines for Drinking-Water Quality, Volume 1: Recommendations. 2nd ed. Geneva, Switzerland:World Health Organization.

[b28-ehp0113-001196] WHO 1996. Guidelines for Drinking-Water Quality, Volume 2: Health Criteria and Other Supporting Information. 2nd ed. Geneva, Switzerland:World Health Organization.

[b29-ehp0113-001196] WHO 1998. Guidelines for Drinking-water Quality, Addendum to Volume 1: Recommendations. 2nd ed. Geneva, Switzerland:World Health Organization.

[b30-ehp0113-001196] WHO 2000. Arsenic Contamination of Drinking Water in Bangladesh. Available: http://www.who.int/peh-super/Oth-lec/Arsenic/Series1/002.htm [accessed 2 May 2000].

[b31-ehp0113-001196] WHO 2001. Arsenic and Arsenic Compounds (EHC 224, 2001). Available: http://www.inchem.org/documents/ehc/ehc/ehc224.htm#1.7 [accessed 22 July 2004].

[b32-ehp0113-001196] WHO 2003. Fact Sheet #210: Arsenic in Drinking Water. Available: http://www.who.int/inf-fs/en/fact210.html [accessed 24 May 2003].

[b33-ehp0113-001196] Yu WH, Harvey CM, Harvey CF (2003). Arsenic in groundwater in Bangladesh: a geostatistical and epidemiological framework for evaluating health effects and potential remedies. Water Res.

